# Seroprevalence of Human T-lymphotropic virus type-1 and -2 infections among first-time United States Blood Donors 2000-2009

**DOI:** 10.1186/1742-4690-11-S1-P61

**Published:** 2014-01-07

**Authors:** Yun Brenda Chang, Zhanna Kaidarova, Daniel Hindes, Marjorie Bravo, Nancy Kiely, Hany Kamel, Denise Dubay, Barbara Hoose, Edward L Murphy

**Affiliations:** 1 Department of Biostatistics, Columbia University, New York, NY, USA; 2 Blood Systems Research Institute, San Francisco, CA, USA; 3 Blood Systems, Inc., Scottsdale, AZ, USA; 4 Departments of Laboratory Medicine and Epidemiology/Biostatistics, University of California San Francisco, San Francisco, CA, USA

## Background

Human T-lymphotropic virus types 1 and 2 (HTLV-1 and –2) are prevalent at low-level among United States blood donors, but recent data on their prevalence is lacking.

## Methods

Data on all first-time blood donors in a large network of United States blood centers was examined during the period 2000-2009. Anti-HTLV-1 and -2 was measured by enzyme immunoassay (EIA) screening with type-specific confirmation by immunofluorescence or RIBA. Prevalence and odds ratios (OR) and 95% confidence intervals (CI) for associations with demographic characteristics were assessed using multivariable logistic regression.

## Results

Among 2,047,740 first-time donors, 104 donors were seropositive for HTLV-I (prevalence 5.1 (95% CI: 4.1 - 6.1) per 100,000) and 300 donors were seropositive for HTLV-2 infection (prevalence 14.7 (95% CI 13.0 - 16.3) per 100,000). Prevalence was lower than reported in the 1990’s but stable from 2000 to 2009. HTLV-1 seropositivity was associated with female sex (OR = 1.56, 95% CI 1.05-2.32); older age; and Black (IR = 25.29, 9% CI 13.14- 48.68) and Asian (OR = 21.43, 95% CI 10.31-44.53) race/ethnicity. HTLV-2 seropositivity was associated with female sex (OR = 2.13, 95% CI 1.67-2.73); older age; and non-white race/ethnicity; residence in the Western (OR=4.12, 95% CI 2.16-7.82) and Southwestern (OR=2.47, 95% CI 1.28-4.78; both vs. Northern) U.S.; and lower educational level.

## Conclusions

HTLV-1 and -2 prevalences among U.S. blood donors declined since the early 1990’s but were stable since 2000. Higher prevalence of HTLV-2 in the West and Southwest may be attributed to endemic foci among Amerindians.

